# Determining the Activity of Imipenem/Relebactam Plus Aztreonam Against Constitutive *Pseudomonas*-Derived Cephalosporinase- and Metallo-β-Lactamase-Producing *Pseudomonas aeruginosa* in a Hollow Fiber Infection Model

**DOI:** 10.1093/ofid/ofag172

**Published:** 2026-03-26

**Authors:** J Nicholas O’Donnell, Kelly E Moolick, Avery I Nahorniak, Maxwell J Gifford, Katherine Young, Thomas P Lodise

**Affiliations:** Department of Pharmacy Practice, Albany College of Pharmacy and Health Sciences, Albany, New York, USA; Department of Pharmacy Practice, Albany College of Pharmacy and Health Sciences, Albany, New York, USA; Department of Pharmacy Practice, Albany College of Pharmacy and Health Sciences, Albany, New York, USA; Department of Pharmacy Practice, Albany College of Pharmacy and Health Sciences, Albany, New York, USA; Department of Infectious Diseases and Vaccines, Merck & Co., Inc., Rahway, New Jersey, USA; Department of Pharmacy Practice, Albany College of Pharmacy and Health Sciences, Albany, New York, USA

**Keywords:** aztreonam, imipenem/relebactam, metallo-β-lactamases, *Pseudomonas aeruginosa*, synergy

## Abstract

**Background:**

Few treatment options exist for serious infections caused by metallo-β-lactamase (MBL)-producing *Pseudomonas aeruginosa*. This study evaluated imipenem/relebactam (I/R) plus aztreonam (ATM) against *Pseudomonas*-derived cephalosporinase (PDC)- and MBL-producing *P. aeruginosa* in a hollow fiber infection model, with ceftazidime/avibactam (CZA) plus ATM as a comparator.

**Methods:**

Two isogenic PDC- and MBL-producing *P. aeruginosa* isolates (MB10480: IMP-1; MB10620: VIM-1) were studied at a starting inoculum of ∼8 log_10_ CFU/mL over 96 hours. Humanized I/R exposures were evaluated alone and with extended-infusion ATM (1.5 g every 6 hours [q6h], 2 g q8h, and 2 g q6h), with CZA plus ATM (2 g q6h) as a comparator. Combinations were tested in triplicate, and ATM/relebactam or ATM/avibactam minimum inhibitory concentrations (MICs) were assessed at 96 hours.

**Results:**

For both MB10480 (ATM/relebactam MIC: 8/4 mg/L) and MB10620 (4/4 mg/L), monotherapy arms mirrored the growth control. Both I/R plus ATM and CZA plus ATM produced early synergy (≥2 log_10_ CFU/mL reduction relative to the most active monotherapy) with >1 log_10_ CFU/mL killing relative to the starting inoculum at 24 hours, followed by regrowth, with bacteriostasis observed at 96 hours across regimens. However, bacterial counts at 96 hours were lowest with I/R plus ATM 2 g q6h. Two colony morphologies were recovered, and retested MICs were occasionally, but not consistently, elevated.

**Conclusions:**

Both I/R plus ATM and CZA plus ATM showed early synergy against PDC- and MBL-producing *P. aeruginosa*, but regrowth occurred with all regimens. Higher-dose ATM with I/R provided more durable suppression, though persistence remained common, underscoring the need to better define dosing and mechanisms of regrowth.

Metallo-β-lactamase (MBL)-producing gram-negative pathogens represent a persistent global public health threat [1]. While several novel β-lactam/β-lactamase inhibitor combinations have been developed to address carbapenem-resistant Enterobacterales, these agents lack intrinsic activity against MBLs and are largely ineffective against MBL-producing *Pseudomonas aeruginosa*, a particularly difficult-to-treat pathogen [2]. This limitation arises because currently available β-lactamase inhibitors do not inhibit MBL enzymes directly. However, therapeutic activity can be restored when these inhibitors are paired with aztreonam (ATM), a monobactam that is inherently stable to hydrolysis by MBLs. In such combinations, the β-lactamase inhibitor protects ATM from degradation by co-produced serine β-lactamases, including extended-spectrum β-lactamases, AmpC enzymes, and serine carbapenemases, thereby enabling effective inhibition of resistant pathogens.

Ceftazidime/avibactam (CZA) plus ATM has emerged as one of the most widely used therapeutic combinations for managing infections caused by MBL-producing Enterobacterales, with efficacy demonstrated in both in vitro and clinical studies [2–5]. However, this combination's activity against MBL-producing *P. aeruginosa* appears more limited, with only modest improvements in susceptibility observed when avibactam (AVI) is combined with ATM, either alone or with conjunction with ceftazidime [3]. This reduced activity may be attributed to resistance mechanisms unique to *P. aeruginosa*, including overexpression of *Pseudomonas*-derived cephalosporinases (PDCs) and active efflux [6]. In this context, relebactam, a diazabicyclooctane β-lactamase inhibitor approved in combination with imipenem/cilastatin, may also exhibit in vitro activity against MBL-producing *P. aeruginosa* paired with ATM. Relebactam retains inhibitory activity against class C PDCs that are not effectively targeted by AVI and is less susceptible to efflux-mediated removal from the periplasmic space [7, 8].

Previous in vitro studies using static concentration time-kill assays have demonstrated synergistic activity between ATM and imipenem/relebactam (I/R) against MBL-producing *P. aeruginosa*, particularly in isolates with OprD porin channel deficiency and constitutive PDC production [9]. Although bacterial killing was most notable at simulated steady-state concentrations, overall reductions in bacterial burden were limited, likely reflecting the limitations of static models that do not replicate the dynamic antimicrobial exposures encountered in vivo. To overcome these limitations, and more accurately simulate human pharmacokinetics, we evaluated the combination of I/R plus ATM using a hollow fiber infection model (HFIM) with humanized exposures. Two previously characterized MBL-producing *P. aeruginosa* isolates were tested to assess the pharmacodynamic activity of the combination and to examine patterns of bacterial killing, regrowth, and development of resistance.

## METHODS

### Bacterial Strains, Antimicrobial Agents, and Susceptibility Testing

Two isogenic, OprD-deficient, *P. aeruginosa* isolates producing both PDC and MBL enzymes were selected (Table 1): *P. aeruginosa* MB10480 (IMP-1) and *P. aeruginosa* MB10620 (VIM-1). Previous studies identified synergy with ATM plus I/R for these isolates in static time-kill studies [9]. Isolates were provided by Merck & Co., Inc. (Rahway, NJ, United States). Imipenem and relebactam powders were supplied by Merck & Co., Inc.; ATM (USP) and ceftazidime (USP) were obtained from Sigma Chemical Co. (St Louis, MO, United States); AVI was obtained from MedChemExpress (Monmouth Junction, NJ, United States). Minimum inhibitory concentrations for I/R, CZA, and ATM were determined via broth microdilution following Clinical and Laboratory Standards Institute guidelines [10]. Minimum inhibitory concentrations for ATM/relebactam were determined using a fixed concentration of relebactam at 4 mg/L. Prior checkerboard studies indicate minimal impact of imipenem presence on ATM/relebactam MIC values [9].

**Table 1.** *Pseudomonas aeruginosa* Isolate Characteristics and Susceptibilities

	MBL	Aztreonam MIC	Imipenem/Relebactam MIC	Ceftazidime/Avibactam MIC	Aztreonam/relebactam MIC	Aztreonam/Avibactam MIC
MB10480	IMP-1	32	128	128	8/4	8/4
MB10620	VIM-1	32	256	128	4/4	8/4

Abbreviations: MBL, metallo-β-lactamase; MIC, minimum inhibitory concentration.

### Hollow Fiber Infection Model

Fresh cation-adjusted Mueller Hinton Broth (CA-MHB; Sigma-Aldrich, St. Louis, MO, United States) was used for all experiments. Hollow fiber infection models were conducted over 96 hours using high-flux polysulfone cartridges (C2011; FiberCell Systems, Frederick, MD, United States), as previously described [11]. Antimicrobial pharmacokinetics were simulated using published human data: half-lives of 1.3 hours for imipenem and relebactam, and 2 hours for ATM and CZA [11–13].

Bacterial isolates were passed on tryptic soy agar (TSA) plus 5% sheep blood plates (Becton Dickinson) for 2 days then allowed to reach log-phase growth in CA-MHB at 37 °C prior to inoculation. Bacterial inocula were prepared according to strain-specific OD_600_ and introduced into the extracapillary space of the HFIM just prior to the start of the model. Initial bacterial inocula of ∼8 log_10_ CFU/mL were used to simulate high-grade ventilator-associated pneumonia [14, 15]. Samples were collected from the infection compartment at 0, 2, 4, 6, 24, 28, 48, 52, 72, and 96 hours for bacterial colony enumeration. Samples were centrifuged at 10 000 *g* for 10 minutes, then reconstituted with sterile CA-MHB to minimize antimicrobial carryover. Bacterial colony quantification was performed in duplicate on LB agar plates (Sigma-Aldrich) using a spiral plater (Interscience SpiralPro, Saint-Nom-la-Bretèche, France), with a lower limit of detection of 2 log_10_ CFU/mL. Cultures were incubated at 37 °C for 18–24 hours before enumeration.

Synergy was defined as ≥2 log_10_ CFU/mL reduction compared to the most active single agent (eg, ATM, I/R, or CZA monotherapy); bactericidal activity was defined as ≥3 log_10_ CFU/mL reduction from the starting inoculum [16]. Isolates recovered at 96 hours were subcultured on TSA with 5% sheep blood for retesting of ATM/relebactam or ATM/AVI MICs.

### Simulated Regimens

Simulated dosing regimens for I/R, CZA, and ATM were based on FDA-approved labels to facilitate clinical translation (Supplementary Table 1) [11, 12, 17–20]. Imipenem/relebactam was simulated as imipenem/cilastatin/relebactam 500/500/250 mg infused over 0.5 hours every 6 hours (q6h) [12]. Cilastatin holds no role in vitro evaluation of imipenem activity, as it inhibits degradation by renal dehydropeptidase in vivo and therefore was not included in this model. Aztreonam dosing regimens (Supplementary Table 1) were adapted from prior work with CZA plus ATM against New Delhi Metallo-β-lactamase-producing Enterobacterales and included simulated doses of 1.5 g q6h, 2 g q8h, and 2 g q6h, each infused over 2 hours [11, 19]. Continuous infusion ATM was excluded due to safety concerns identified in a recent phase I study [21]. All combination regimens were administered with simultaneous start times for I/R and ATM or CZA and ATM. Combination regimens were performed in triplicate; monotherapy arms (ATM, I/R, and CZA) were evaluated in singlet, as killing was not anticipated and variability in nonkilling conditions was expected to be low.

Because ATM has a longer half-life than I/R, supplemental ATM dosing was delivered between scheduled infusions using programmable syringe pumps (New Era Pump Systems, Farmingdale, NY, United States). Supplemental regimens were designed using Pmetrics for R (R Core Team, Vienna, Austria) [22].

For comparative purposes, CZA (2.5 g q8h; 2-hour infusion) plus ATM (2 g q6h; 2-hour infusion) was also simulated, based on prior optimization studies of this combination against MBL-producing Enterobacterales [11].

### Antibiotic Assay Methods

Pharmacokinetic validation samples were drawn from the central compartment at 0.5 (I/R regimens only), 2, 4, 6, 24, 27, and 30 hours. Samples were stabilized with a solution of 1 M MES (chemical name, 2-(N-Morpholino)ethanesulfonic acid [C_6_H_13_NO_4_S]), ethylene glycol, and methanol (2:1:1:4 v/v) and stored at −80 °C until being assayed [23].

### Imipenem, Relebactam, Aztreonam, and Avibactam

Cation-adjusted Mueller Hinton Broth, preservative, acetonitrile, and a 10 mM MES buffer (pH 6.7) in acetonitrile/water (50/50, prepared by dilution of 1 M MES buffer, pH 6.7, 0.384 g MES, and 1.74 g MES sodium salt in 10 mL water) were used in appropriate ratios during sample preparation to ensure matrix matching of the prepared unknown samples, standards, quality controls, as well as single and double blanks. Protein precipitation was performed by the addition of a 4:1 ratio of methanol to the matrix-matched samples. Following centrifugation, 250 µL supernatant was transferred to a clean plate for liquid chromatography–mass spectrometry (LC-MS) analysis.

Liquid chromatography–tandem mass spectrometry (LC-MS/MS) analysis was performed on a Waters Acquity ultra-performance liquid chromatography system interfaced to an AB Sciex 4500 mass spectrometer utilizing the turbo ion spray interface operated under positive ionization mode. Separation of imipenem, relebactam, and AVI was achieved on a HALO HILIC column (150 × 4.6 mm, 2.7 µm) using gradient elution with mobile phases consisting of 10 mM ammonium formate in water (mobile phase A) and 10 mM ammonium formate in acetonitrile (mobile phase B) at a flow rate of 1 mL per minute. The chromatography was run using the following conditions: the column was initially equilibrated with 10% A; after sample injection, 10% solvent A was maintained for 0.5 minute before it was increased linearly to 95% solvent A over a 0.5 minute period. The column was maintained at 95% solvent A for 1.3 minutes and then returned to the initial conditions. The column was then fully equilibrated for 0.80 minute. The total analytical time was 3.20 minutes. Quantifications were completed by monitoring the transition of *m*/*z* 300 to *m*/*z* 98 for imipenem, *m*/*z* 349 to *m*/*z* 269 for relebactam, *m*/*z* 436 to *m*/*z* 313 for ATM, and *m*/*z* 266 to *m*/*z* 186 for AVI. All concentrations were determined by weighted (1/×2) linear regression of the standard curves.

### Ceftazidime

Samples were prepared in a matrix of CA-MHB, followed by the addition of stabilizer solution (1:1 ethylene glycol and 1 M MES) and methanol in a 1:1:2 ratio (2:1:1:4 for sample, ethylene glycol, MES, and methanol). Plates were prepared for LC-MS/MS analysis by aliquoting 20.0 µL of each unknown sample, standard, and quality control into 500 µL 300 ng/mL ceftazidime-d5 in water. Plates were then capped, vortexed for 10 minutes, and transferred to the autosampler for analysis. The autosampler temperature was set at 4 °C for analysis.

All samples were assayed via LC-MS/MS on an ExionLC AC ultra-high-performance LC system coupled to an AB Sciex 5500 mass spectrometer. A binary gradient mobile phase consisting of ammonium formate (20 mM NH_4_HCO_2_; pH 4.3) and methanol was used (Supplementary Table 2), with a Synergi Polar RP column (80A, 50 × 3 mm, 4 µm particles). Column temperature was set at 35 °C, sample injection volume was 1 µL, flow rate was set at 0.5 mL/min, and total run time was 3.5 minutes. Quantification was completed by monitoring the transition of *m*/*z* 547.2 to *m*/*z* 486.1. The calibration curve ranged from 2.5 to 150 µg/mL (*R*^2^: 0.9997–0.9998). All concentrations were determined by weighted (1/×2) linear regression of the standard curves.

### Pharmacokinetic Analysis of Hollow Fiber Infection Model Concentrations

Antimicrobial exposures were validated by comparison with simulations derived from previously published population pharmacokinetic models for each agent using Pmetrics for R [22]. For each drug, 1000 patient profiles were simulated based on published data in patients with normal renal function [11, 12, 17–19]. Accordingly, simulated exposures represent conservative estimates relative to those expected in patients with renal dysfunction. Observed concentrations in the hollow fiber model were compared with simulated median and 95% confidence interval profiles for each regimen.

In-run validation assays for imipenem and ceftazidime were inconsistent with separate pharmacokinetic validation runs performed using identical preparation and administration methods. Imipenem concentrations were lower than expected, potentially due to postsampling degradation or hydrolysis by β-lactamases within the infection compartment. In contrast, observed ceftazidime peak concentrations (>200 mg/L) exceeded plausible levels based on dosing syringe contents, suggesting assay interference or carryover; such concentrations would require infusion of nearly the entire 24-hour dosing syringe during a single dosing interval. Because drug-only validation runs without bacteria demonstrated accurate pharmacokinetics, these anomalies were unlikely to reflect true exposures in the experiments, and the corresponding HFIM data were retained for analysis (Supplementary Figure 1). Additionally, AVI concentrations were modestly higher than predicted, which would be expected to bias results in favor of the CZA plus ATM regimen. Model fits for the remaining agents were satisfactory (Supplementary Figure 2).

### Comparisons of Bacterial Killing

Endpoints included change in bacterial burden at 24 and 96 hours relative to baseline, and the integrated area under the bacterial kill curve (AUBKC) over 96 hours. AUBKC was calculated using the linear trapezoidal rule. Comparative analysis of AUBKC among regimens was performed using 1-way analysis of variance with post hoc Tukey HSD correction for multiple comparisons with Intercooled Stata v15.1 (StataCorp, College Station, TX, United States).

## RESULTS

### Isolate Susceptibility

#### MB10480 (IMP-1 Producer)

MB10480 exhibited in vitro resistance to ATM, I/R, and CZA, as expected (Table 1). The MIC for ATM was 32 mg/L, while the MICs for I/R and CZA were both 128 mg/L. The MICs for ATM in combination with relebactam (ATM/R) and ATM/AVI were 8/4 mg/L.

#### MB10620 (VIM-1 Producer)

Similar results were observed for MB10620, with an ATM MIC of 32 mg/L (Table 1). The ATM/R and ATM/AVI MICs were 4/4 and 8/4 mg/L, respectively.

### Hollow Fiber Infection Model Experiments

#### MB10480

**Figure 1.**
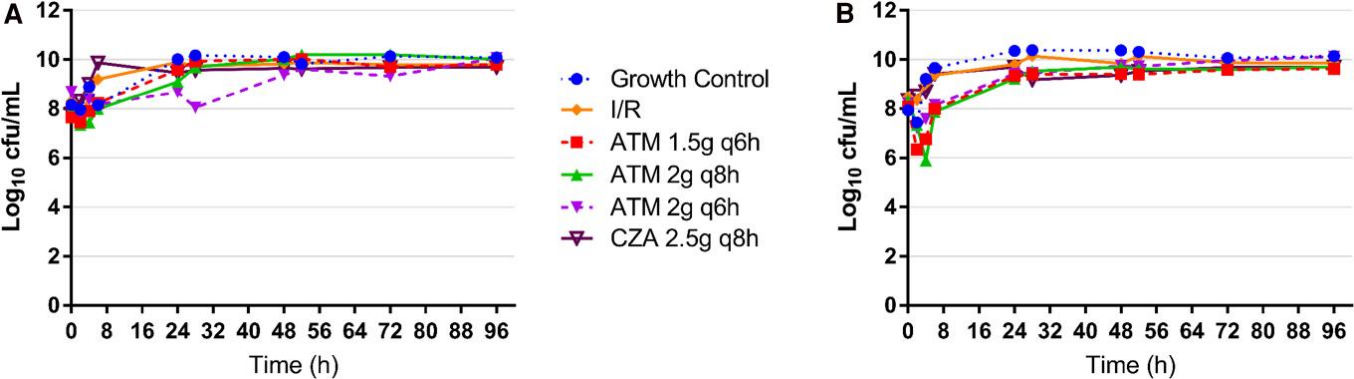
Hollow fiber infection models evaluating bacterial killing of monotherapy regimens against *Pseudomonas aeruginosa* (*A*) MB10480 and (*B*) MB10620.

**Figure 2.**
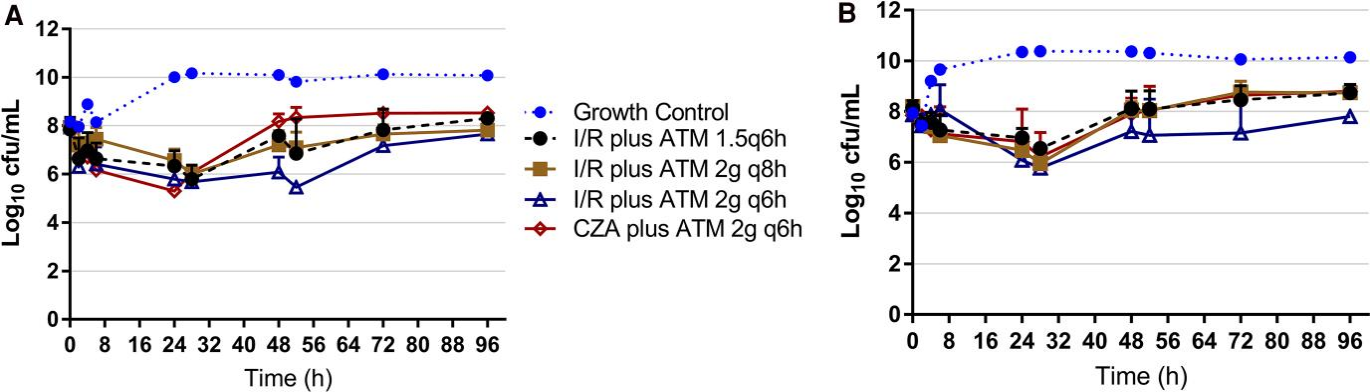
Hollow fiber infection models evaluating bacterial killing of combination regimens against *Pseudomonas aeruginosa* (*A*) MB10480 and (*B*) MB10620.

All monotherapy regimens resulted in bacterial growth comparable to the growth control, with the exception of ATM 2 g q6h, which demonstrated initial inhibition of bacterial growth through 24 hours (Figure 1*A*). However, regrowth occurred thereafter, returning to levels similar to the growth control. All combinations of I/R plus ATM resulted in bacterial counts similar to the starting inoculum by 96 hours, which was consistent across all ATM dosing regimens (Figure 2*A*). Replicate variability was minimal across all I/R plus ATM and CZA plus ATM combinations (Supplementary Figures 3 and 4).

At 24 hours, all combination regimens demonstrated synergy (≥2 log_10_ CFU/mL reduction relative to the most active individual agent), achieving an average of 1.37–2.65 log_10_ CFU/mL reduction relative to the starting inoculum (Table 2). Regrowth was observed with all regimens by 96 hours. Among them, only I/R plus ATM 2 g q6h maintained bacterial counts below the starting inoculum throughout the experiment. This regimen achieved −2.29 log_10_ CFU/mL reduction at 24 hours and −0.43 log_10_ CFU/mL by 96 hours. The I/R plus ATM 1.5 g q6h regimen achieved −1.52 log_10_ CFU/mL at 24 hours but regrew to 8.45 log_10_ CFU/mL by 96 hours. Similarly, I/R plus ATM 2 g q8h achieved −1.36 log_10_ CFU/mL killing at 24 hours, followed by regrowth to near baseline (−0.11 log_10_ CFU/mL). Ceftazidime/avibactam plus ATM 2 g q6h achieved comparable 24-hour killing (−2.65 log_10_ CFU/mL) but regrew slightly above baseline by 96 hours (0.57 log_10_ CFU/mL relative to starting inoculum).

**Table 2.** Summary of Bacterial Killing

	MB10480			MB10620		
	Change Relative to Starting Inoculum at 24 h	Change Relative to Starting Inoculum at 96 h	AUBKC_0–96h_	Change Relative to Starting Inoculum at 24 h	Change Relative to Starting Inoculum at 96 h	AUBKC_0–96h_
Growth control	1.860	1.936	…	2.421	2.215	…
I/R	1.582	1.501	…	1.333	1.391	…
ATM 1.5 g q6h	1.937	2.151	…	1.253	1.545	…
ATM 2 g q8h	1.102	2.028	…	0.904	1.387	…
ATM 2 g q6h	−0.013	1.381	…	1.205	1.908	…
I/R plus ATM 1.5 g q6h	−1.516^a^	0.454	677.16^b^	−1.173^a^	0.610	751.93^b^
I/R plus ATM 2 g q8h	−1.366^a^	−0.119^a^	689.40^b^	−1.725^a^	0.548	742.60^b^
I/R plus ATM 2 g q6h	−2.295^a^	−0.431^a^	619.13	−1.783^a^	−0.071^a^	677.84
CZA	1.669	1.902	…	1.349	1.360	…
CZA plus ATM 2 g q6h	−2.647^a^	0.573	716.23^b^	−1.022^a^	0.963	746.56^b^

Abbreviations: ATM, aztreonam; AUBKC, area under the bacterial kill curve; CZA, ceftazidime/avibactam; I/R, imipenem/relebactam; q6h, every 6 hours; q8h, every 8 h.

^a^Synergistic (ie, >2 log_10_ CFU/mL relative to most active individual agent).

^b^ *P* < .05 for comparison with I/R plus ATM 2 g q6h.

Across all combination regimens, regrowth appeared to be driven by the emergence of small-colony variants (SCVs). Resistance development, defined as a >3-fold increase in MIC from baseline, was inconsistently observed. Among SCVs recovered from I/R plus ATM regimens, resistance developed in 3 of 9 isolates; no resistance was detected in typical morphology colonies. For CZA plus ATM, resistance emerged in 2 of 3 typical isolates and 1 of 3 SCVs (Table 3).

**Table 3.** Aztreonam/Relebactam or Aztreonam/Avibactam MIC Results from Isolates Recovered at 96 Hours

Simulated Regimen	Replicate	Morphology	MB10480	MB10620
			MIC (mg/L)	MIC (mg/L)
I/R 0.5 g/0.25 g q6h (0.5 h infusion) plus ATM 1.5 g q6h (2 h infusion)^a^	1	Typical	4/4	32/4^b^
		SCV	16/4	32/4^b^
	2	Typical	4/4	8/4
		SCV	8/4	16/4^b^
	3	Typical	4/4	4/4
		SCV	4/4	8/4
I/R 0.5 g/0.25 g q6h (0.5 h infusion) plus ATM 2 g q8h (2 h infusion)^a^	1	Typical	4/4	16/4^b^
		SCV	32/4^b^	8/4
	2	Typical	4/4	8/4
		SCV	16/4	8/4
	3	Typical	8/4	4/4
		SCV	16/4	32/4^b^
I/R 0.5 g/0.25 g q6h (0.5 h infusion) plus ATM 2 g q6h (2 h infusion)^a^	1	Typical	4/4	16/4^b^
		SCV	8/4	16/4^b^
	2	Typical	4/4	8/4
		SCV	64/4^b^	8/4
	3	Typical	8/4	32/4^b^
		SCV	32/4^b^	64/4^b^
CZA 2 g/0.5 g q8h (2 h infusion) plus ATM 2 g q6h (2 h infusion)^c^	1	Typical	8/4	16/4
		SCV	16/4	16/4
	2	Typical	32/4^b^	32/4^b^
		SCV	32/4^b^	16/4
	3	Typical	32/4^b^	4/4
		SCV	16/4	4/4

Abbreviations: ATM, aztreonam; CZA, ceftazidime/avibactam; I/R, imipenem/relebactam; MIC, minimum inhibitory concentration; q#h, every # hours; SCV, small-colony variant.

^a^MICs are aztreonam/relebactam.

^b^MIC >3-fold higher than parent isolate.

^c^MICs are aztreonam/avibactam.

#### MB10620

As with MB10480, monotherapy regimens showed no significant bacterial killing (Figure 1*B*). All combination regimens exhibited bacterial counts similar to the starting inoculum by 96 hours, though only I/R plus ATM 2 g q6h maintained counts below the starting inoculum (−0.07 log_10_ CFU/mL; Figure 2*B*). Again, minimal variability was observed across replicates (Supplementary Figures 3 and 4).

All combination regimens demonstrated synergy at 24 hours, followed by regrowth (Table 2). The greatest 24-hour killing occurred with I/R plus ATM 2 g q6h and 2 g q8h, achieving reductions of −1.78 and −1.72 log_10_ CFU/mL, respectively. Imipenem/relebactam plus ATM 1.5 g q6h achieved ∼1 log_10_ CFU/mL reduction at 24 hours, followed by regrowth above baseline by 96 hours (0.61 log_10_ CFU/mL). Regrowth was also observed with ATM 2 g q8h and 2 g q6h combinations (0.54 and −0.07 log_10_ CFU/mL at 96 hours, respectively). Ceftazidime/avibactam plus ATM reduced counts by −1.02 log_10_ CFU/mL at 24 hours, followed by regrowth to 0.96 log_10_ CFU/mL at 96 hours. Notably, I/R plus ATM 2 g q6h was the only regimen that maintained synergy through 96 hours.

As with MB10480, regrowth appeared to be SCV driven. Resistance emerged more frequently in MB10620 than in MB10480. Among SCVs recovered from I/R plus ATM regimens, resistance developed in 5 of 9 isolates. Resistance also emerged in 4 of 9 typical morphology isolates. In contrast, resistance was detected in only one typical morphology isolate in the CZA plus ATM group, and none of the SCVs (Table 3).

#### Comparisons of Bacterial Killing

For both isolates, the AUBKC over 96 hours was significantly lower with I/R plus ATM 2 g q6h compared with I/R plus lower-dose ATM regimens and CZA plus ATM (*P* < .05 for all comparisons; Table 2).

## DISCUSSION

To our knowledge, this is the first study to evaluate the combination of I/R and ATM against MBL-producing *P. aeruginosa* using a dynamic in vitro infection model. This platform is frequently employed to simulate clinically relevant pharmacokinetic exposures and to identify effective combination regimens for multidrug-resistant gram-negative organisms [24]. Therapeutic options for infections caused by these highly drug-resistant pathogens remain extremely limited [2]. Although ATM may retain activity against MBL-producing *P. aeruginosa* in the absence of chromosomal AmpC (ie, PDC) β-lactamase overexpression and without co-production of additional β-lactamases, such susceptible resistance profiles are increasingly uncommon among contemporary clinical isolates [3, 25]. Cefiderocol has demonstrated in vitro potency against MBL producers; however, elevated MICs are frequently observed among strains harboring IMP-type MBLs, which can further restrict treatment options [26]. Polymyxins may also retain in vitro activity, yet their clinical utility is constrained by nephrotoxicity, limited penetration into critical infection sites such as the lung, and inconsistent clinical efficacy [27, 28]. In this context, the combination of I/R plus ATM warrants investigation as a potentially viable therapeutic strategy, particularly given the fatal burden of MBL-producing *P. aeruginosa* in nosocomial infections worldwide [1].

Several key findings emerged from this study. During the first 24 hours, both I/R plus ATM and CZA plus ATM produced similar early antibacterial effects, achieving ∼1–2 log_10_ CFU/mL reductions in bacterial density. However, important differences emerged in the durability of suppression and patterns of resistance. Although regrowth ultimately occurred with all regimens, I/R plus ATM with the highest ATM exposure (2 g q6h) achieved greater and more sustained suppression below the starting inoculum and was the only regimen that consistently maintained bacterial counts below baseline through 96 hours. Consistent with this, the AUBKC over 96 hours was significantly lower with I/R plus ATM, particularly with ATM 2 g q6h, compared with CZA plus ATM, indicating superior overall bacterial suppression across the dosing interval. Despite these differences in durability, regrowth was observed with all regimens by the end of the experiment. Importantly, resistance emergence was inconsistent and did not fully explain the observed bacterial recovery. Nonetheless, resistance among typical morphology isolates was more frequent with CZA plus ATM than with I/R plus ATM (3/6; 50% vs 4/18; 22%), suggesting that I/R plus ATM may better suppress dominant, rapidly growing populations even when persistence ultimately occurs. Instead, the predominant mechanism of regrowth appeared to be phenotypic adaptation, as evidenced by the frequent emergence of SCVs, rather than stable acquired genotypic resistance. Pharmacokinetic validation identified slightly higher than predicted AVI concentrations, which would be expected to bias results in favor of CZA plus ATM, rendering our comparative findings conservative with respect to any advantage of I/R plus ATM. These observations are consistent with prior dynamic model studies. For example, Noel and colleagues demonstrated >4 log_10_ CFU/mL killing with simulated I/R exposures followed by regrowth by day 7 in the absence of detectable resistance, with resistant subpopulations emerging only with longer (14-day) selective pressure [13]. In contrast, similar HFIM studies of I/R or CZA plus ATM against MBL-producing Enterobacterales have not shown comparable regrowth over similar time frames, suggesting that the adaptive regrowth observed here may reflect species-specific persistence mechanisms unique to *P. aeruginosa* [11].

In our study, regrowth was primarily associated with the emergence of SCVs, a phenotype particularly evident in simulations involving lower-dose ATM regimens. Small-colony variants are characterized by altered metabolic activity, reduced growth rates, and enhanced tolerance to antibiotics, particularly to cell wall–active agents such as ATM that target penicillin-binding protein 3 (PBP3) [29–31]. These traits, along with a propensity to persist within biofilm-like microenvironments, likely contribute to survival under sustained β-lactam exposure [32–34]. Our findings align with the study by Smith et al, who reported a non-culturable, filamentous phenotype in a well-characterized VIM-2–producing *P. aeruginosa* isolate following ATM exposure in an HFIM [35]. In contrast, the SCVs recovered in our study remained culturable, suggesting strain-specific or model-dependent differences in adaptive responses to ATM-based therapy. Variation in experiment duration between the presented data and that of Smith et al (96 hours compared with 168 hours) may account for differences in observed phenotype development. Given our findings and prior reports linking ATM exposure to SCV emergence, the association between SCV development and ATM-mediated PBP3 inhibition warrants further mechanistic investigation. One possibility is that ATM-induced stress on PBP3 function activates global stress response pathways, such as the SOS or stringent response, which have been implicated in SCV formation and persistence [36–38]. In addition, mutations or regulatory changes in genes involved in oxidative stress tolerance, electron transport, or quorum sensing may contribute to the SCV phenotype as a survival strategy under antimicrobial pressure [39–41]. Further studies are needed to elucidate the specific mechanisms underlying SCV emergence in this context.

This study has several important limitations. Only 2 isogenic *P. aeruginosa* isolates with relatively high ATM/relebactam MICs (4–8 mg/L) were included, which limits the generalizability of these findings to other MBL-producing strains, particularly those exhibiting higher-level resistance to I/R plus ATM. These isolates were selected based on previously published checkerboard and time-kill experiments suggesting synergistic effect and therefore may provide an optimistic estimation of bacterial effect for other MBL-producing *P. aeruginosa*. Furthermore, the hollow fiber cartridges utilized in these experiments are known to retain exogenous β-lactamases released from lysed bacterial cells, which may have influenced the pharmacodynamic activity of the combination regimens over time. Although these enzymes gradually degrade, their retention could contribute to underestimation of bacterial killing and overestimation of regrowth potential in the infection compartment. While assayed concentrations were generally within target ranges in the central compartment, separate validation of the extra-cellular compartment of the hollow fiber was not conducted. Based on previously published data showing bacterial killing of β-lactam agents using the C2011 cartridge, we do not expect concentrations to differ significantly from central compartment concentrations [42–45]. In addition, we did not characterize the fitness, virulence attributes, or growth kinetics of recovered SCVs, limiting our ability to predict their clinical relevance and the potential impact on treatment outcomes. Finally, optimal pharmacodynamic targets for relebactam exposure in combination with ATM against MBL-producing *P. aeruginosa* remain undefined. Higher inhibitor concentrations may be necessary to achieve sustained suppression of bacterial regrowth and to prevent the emergence of adaptive phenotypes.

In summary, the combination of I/R plus ATM demonstrated synergistic activity against MBL-producing *P. aeruginosa* in a dynamic in vitro infection model. The high-dose ATM regimen (2 g q6h) provided the most durable bacterial suppression over 96 hours. However, SCV-associated regrowth occurred across all regimens, underscoring the capacity of *P. aeruginosa* to adopt phenotypic adaptations that facilitate persistence in the presence of potent β-lactam combination therapy. Future studies are needed to further define the clinical relevance of SCV emergence, elucidate pharmacodynamic targets for relebactam in combination regimens, and characterize the role of exogenous β-lactamases and other adaptive mechanisms in driving bacterial regrowth during prolonged treatment.

## Supplementary Material

ofag172_Supplementary_Data

## Supplementary Data

Supplementary materials are available at *Open Forum Infectious Diseases* online. Consisting of data provided by the authors to benefit the reader, the posted materials are not copyedited and are the sole responsibility of the authors, so questions or comments should be addressed to the corresponding author.
