# Supplementary material for: Determining the Activity of Imipenem/Relebactam Plus Aztreonam Against Constitutive Pseudomonas-Derived Cephalosporinase- and Metallo-β-Lactamase-Producing Pseudomonas aeruginosa in a Hollow Fiber Infection Model
Source: Open Forum Infect Dis. 2026 Mar 26;13(4):ofag172. doi: 10.1093/ofid/ofag172 (PMC13102097; doi:10.1093/ofid/ofag172)

**Determining the Activity of Imipenem/Relebactam Plus Aztreonam Against Constitutive Pseudomonas-Derived Cephalosporinase- and Metallo-β-Lactamase-Producing *Pseudomonas aeruginosa* in a Hollow Fiber Infection Model**

**Supplemental** **Table 1. Simulated Regimens**

| **Regimen** | **Imipenem/ Relebactam Regimen** | **Aztreonam Regimen** |
| --- | --- | --- |
| Control | n/a | n/a |
| I/R Monotherapy | 0.5/0.25g q6h (0.5 h infusion) | n/a |
| ATM 1.5g q6h Monotherapy | n/a | 1.5 g q6h (2-hour infusion) |
| ATM 2g q8h Monotherapy | n/a | 2 g q8h (2-hour infusion) |
| ATM 2g q6h Monotherapy | n/a | 2 g q6h (2-hour infusion) |
| I/R plus ATM 1.5g q6h | 0.5/0.25g q6h (0.5 h infusion) | 1.5 g q6h (2-hour infusion) |
| I/R plus ATM 2g q8h | 0.5/0.25g q6h (0.5 h infusion) | 2 g q8h (2-hour infusion) |
| I/R plus ATM 2g q6h | 0.5/0.25g q6h (0.5 h infusion) | 2 g q6h (2-hour infusion) |
|  | **Ceftazidime/Avibactam Regimen** | **Aztreonam Regimen** |
| CZA Monotherapy | 2/0.5 g q8h (2-hour infusion) | n/a |
| CZA plus ATM | 2/0.5 g q8h (2-hour infusion) | 2 g q6h (2-hour infusion) |

**Supplemental Table 2. Gradient method for ceftazidime analysis**

| **Time (min)** | **%B** |
| --- | --- |
| **0.0** | **20** |
| **0.5** | **75** |
| **2** | **70** |
| **2.5** | **20** |
| **3.5** | **20** |

Supplemental Figure 1. Target and Observed Antimicrobial Concentrations for Imipenem and Ceftazidime in Sterile Pharmacokinetic Validation (a, b) and In-Run Pharmacokinetic Validation (c, d)


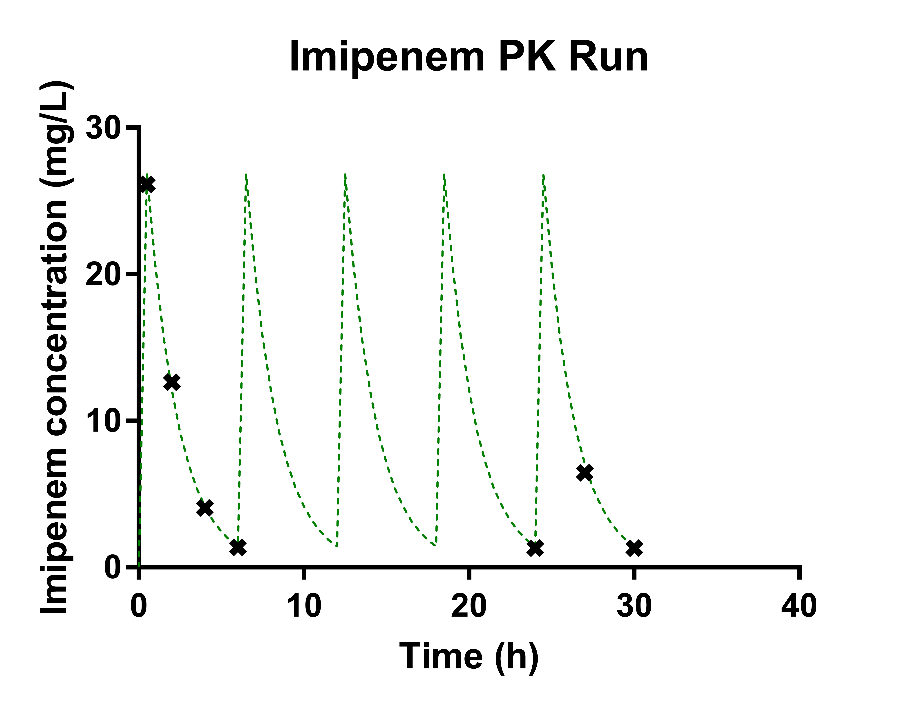

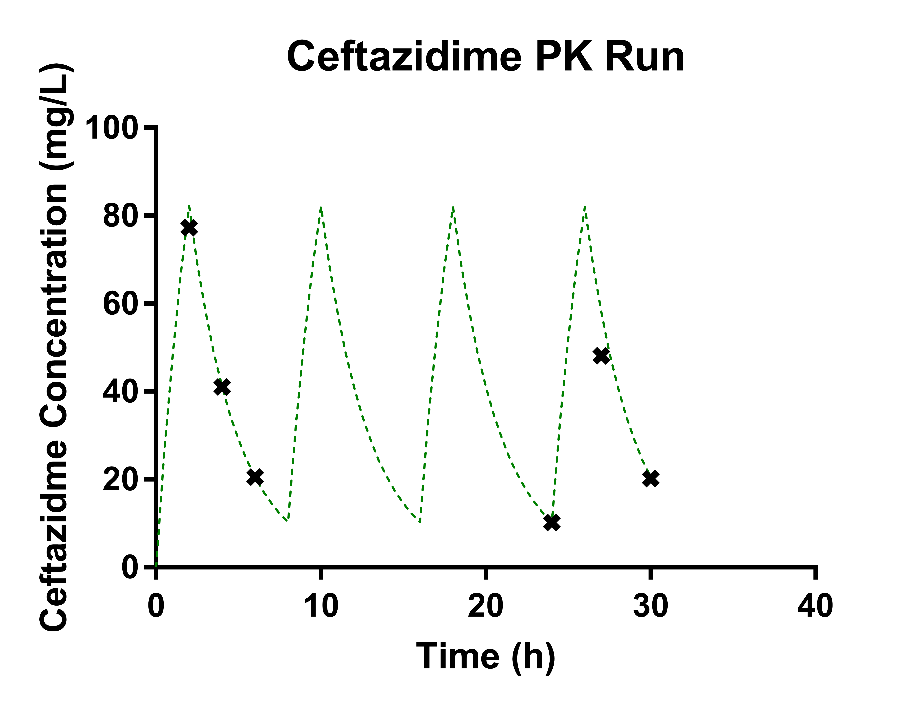


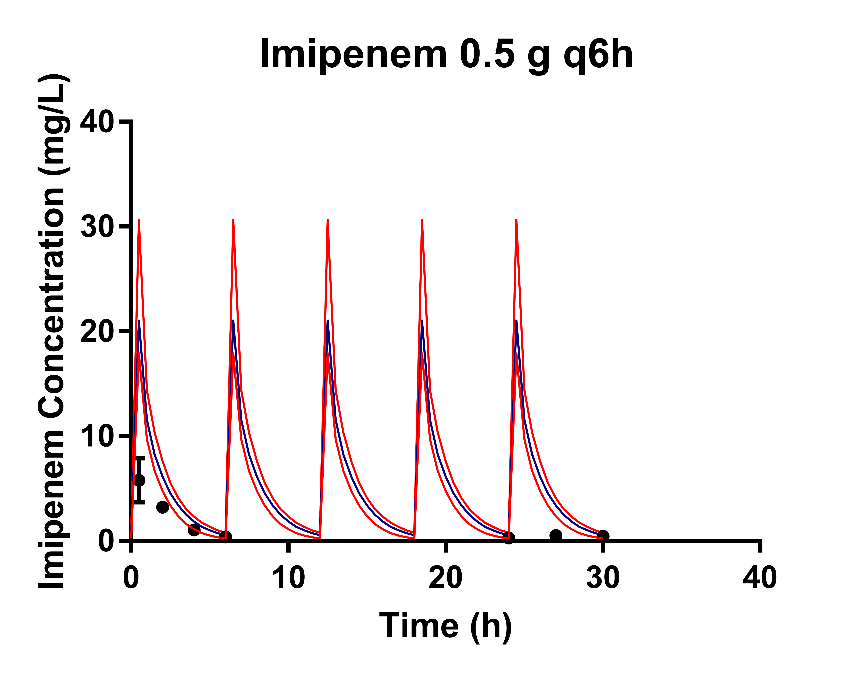

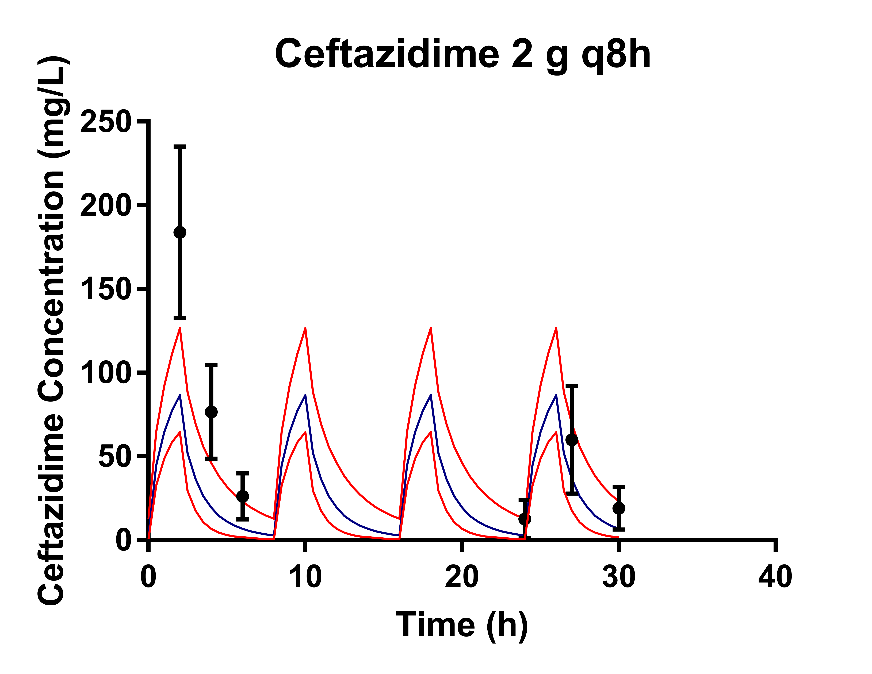


Dotted line represents expected concentrations in the HFIM; Xs represent observed concentrations

Supplemental Figure 2. Observed In-run Antimicrobial Concentrations for Aztreonam 1.5 g q6h (a), 2 g q8h (b), 2g q6h (c), relebactam 0.25 g q6h (d), and avibactam 0.5 g q8h (e)


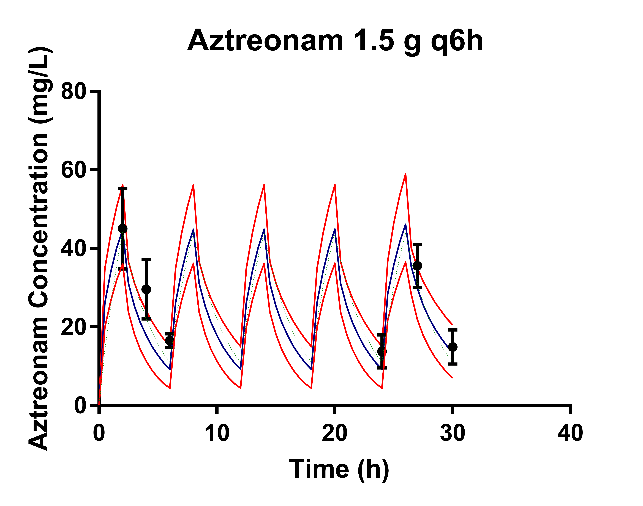

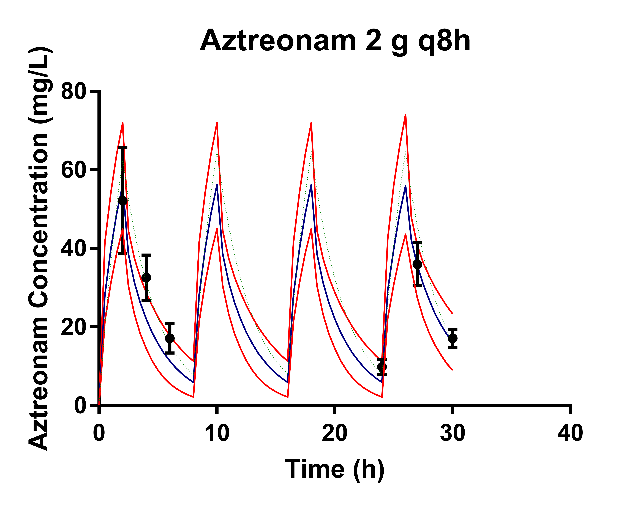

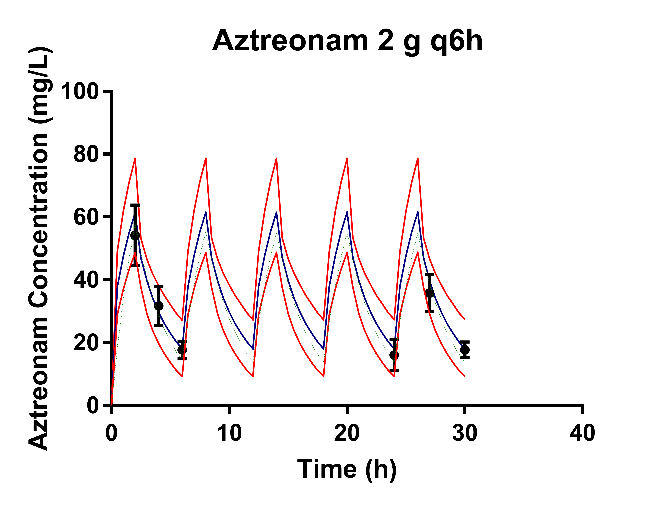

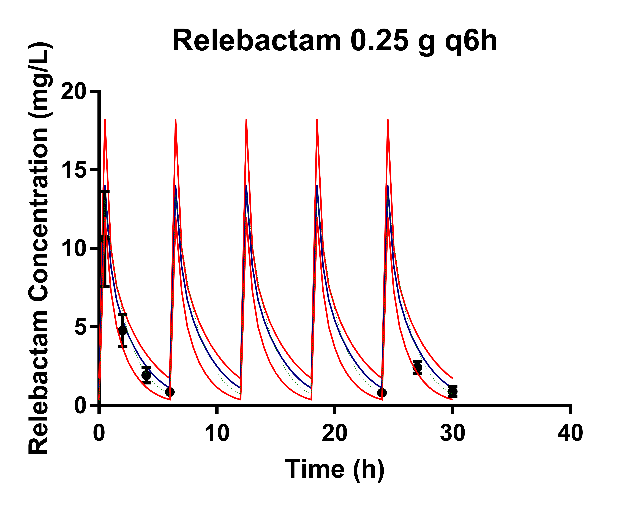

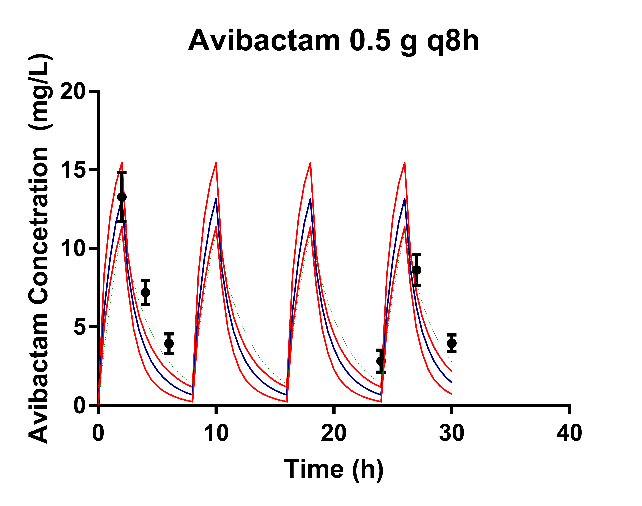


Circles and bars represent the mean and standard deviation; solid red bars indicate the 5 and 95% confidence interval bands, solid blue line indicates median exposure, dotted green line indicates predicted exposures

Supplemental Figure 3. Replicates of hollow fiber runs for I/R plus ATM 1.5g q6h (a, d), I/R plus ATM 2g q8h (b, e), and I/R plus ATM 2g q6h (c, f)


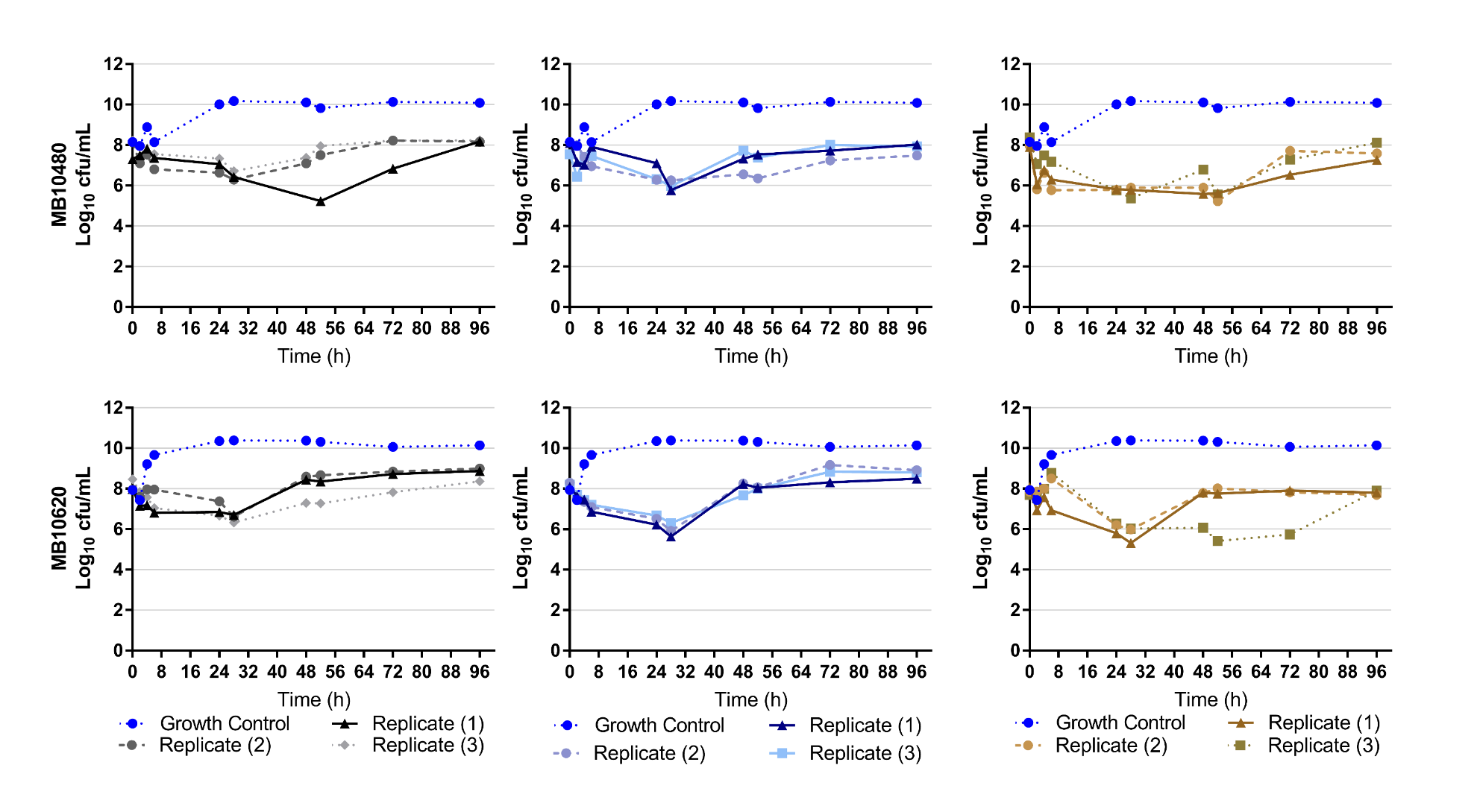


Supplemental Figure 4. Replicates of hollow fiber runs for CZA plus ATM 2g q6h against MB10480 (a) and MB10620 (b


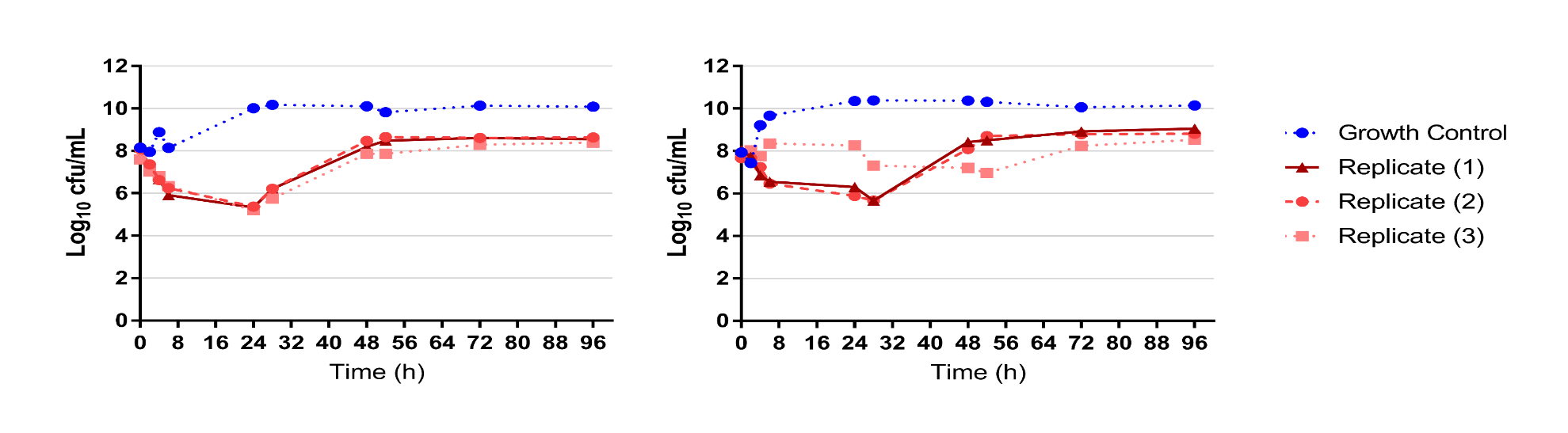

Supplement: ofag172_Supplementary_Data [file ofag172_supplementary_data.docx]
